# Accurate and precise determination of ^90^Sr at femtogram level in IAEA proficiency test using Thermal Ionization Mass Spectrometry

**DOI:** 10.1038/s41598-019-52890-3

**Published:** 2019-11-11

**Authors:** Norbert Kavasi, Sarata Kumar Sahoo, Hideki Arae, Tatsuo Aono, Zenon Palacz

**Affiliations:** 1 Environmental Radionuclide Research Group, Centre for Advanced Radiation Emergency Medicine, National Institutes for Quantum and Radiological Science and Technology (QST) 4-9-1, Anagawa, Inage-ku, Chiba 263-8555 Japan; 2 IsotopX Ltd, Middlewich, Cheshire CW 10 0HU United Kingdom

**Keywords:** Mass spectrometry, Environmental chemistry

## Abstract

A novel method for the determination of ultra-trace level ^90^Sr has been recently developed applying thermal ionization mass spectrometry (TIMS). The method includes the chemical separation of Zr (isobaric interference of ^90^Zr) from the samples followed by determination of ^90^Sr/^88^Sr abundance  sensitivity (2.1 × 10^−10^). The analytical performance of this method was assessed in the IAEA-TEL 2017-3 worldwide open proficiency test. For ^90^Sr determination, tap water and milk powder samples were distributed amongst the participant laboratories with reference values of 11.2 ± 0.3 Bq kg^−1^ (2.2 ± 0.1 fg g^−1^) and 99.9 ± 5.0 Bq kg^−1^ (19.5 ± 1.0 fg g^−1^), respectively. The stable Sr concentrations were 39.4 ± 0.9 ng g^−1^ and 2.5 ± 0.1 µg g^−1^ while the ^90^Sr/^88^Sr isotope ratios were 6.47 ± 0.17 × 10^−8^ and 9.04 ± 0.45 × 10^−9^ in the tap water and milk powder samples, respectively. For TIMS measurement, 50 mL water and 1 g milk powder samples were taken for analysis. This TIMS method demonstrated an impressive accuracy (relative bias of 4.2% and −2.1%, respectively) and precision (relative combined uncertainty of 4.1% and 7.6%, respectively) when compared with radiometric techniques. For the first time in the history of inorganic mass-spectrometry, ^90^Sr analysis using a TIMS instrument is confirmed by an independent proficiency test.

## Introduction

The ^90^Sr is an artificial isotope of strontium (Sr) whereas natural Sr comprises of four stable isotopes such as, ^84^Sr, ^86^Sr, ^87^Sr and ^88^Sr. ^88^Sr has the highest abundance of 82.58%. The ^90^Sr (*T*_*1/2*_ = 28.8 y) is a pure beta emitter, produced by the fission of U and Pu isotopes. Low level (some Bq kg^−1^ or mBq kg^−1^) ^90^Sr contamination exists in different environmental matrices as a result of nuclear weapon tests and nuclear accidents. Basically, Sr element has no biological role in the human body, however it has the potential to incorporate into the bone structure because it is bio-chemically similar to calcium, thus the ^90^Sr isotope can cause long-term radiation dose. From the view point of public health and natural radiation protection, precise and accurate determination of ^90^Sr in various environmental, biological and radioactive waste samples is essential^1^.

In general, radiometric methods have been mainly used for ^90^Sr analysis, where beta particles are emitted by the radioactive decay of ^90^Sr which are detected using different types of detectors, such as, gas ionization detector, solid or liquid scintillators, etc. In recent years, owing to significant developments on sample introduction, detector and interference removal techniques, many successful ^90^Sr determinations have been reported using mass spectrometry instruments. For this purpose, inductively coupled plasma mass spectrometry (ICP-MS) instruments with collision/reaction cells or triple quadrupole system have been primarily applied^2–5^. Alternatively, thermal ionisation mass spectrometry (TIMS) is a robust technique for ^90^Sr analysis^6^. The main advantages of the mass spectrometry method over the radiometric are the shorter analysis time, higher sample throughput and smaller sample intake.

The low level ^90^Sr detection in environmental samples with mass spectrometry instrument is a challenging task. The first critical point is the isobaric interference of ^90^Zr as a consequence of significant amounts of Zr element in environmental samples. For example, in case of Japanese soil samples, the mean concentration of Zr element is around 90 µg g^−1^ (varying in the range from 0.6 to 430 µg g^−1^)^7^, which corresponds to ~46 µg g^−1^ of ^90^Zr since its isotopic abundance is 51.5%^8^. The typical ^90^Sr level in environmental samples is quite low, in the range of fg g^−1^, for contaminated Fukushima soil samples it is around 4 fg g^−1^ (20 Bq kg^−1^) or lower^9^. In order to successfully measure ^90^Sr using inorganic mass spectrometry either a high mass resolution (~30,000) which is not yet available to resolve the isobaric ^90^Zr peak or an efficient chemical purification method for removal of Zr from Sr at ultra-trace levels is required^10,11^. For practical purposes, quite a high decontamination factor (in the range of 10^8^ to 10^11^) is required for ^90^Zr isobaric interference removal (accepting just a 10% contribution to the ^90^Sr signal) in case of soil samples^12^.

The second critical point for the application of mass spectrometry instruments for ^90^Sr determination is the peak tailing on the higher mass side from ^88^Sr. Although there exists reliable ultra-high vacuum condition (<10^−8^ Pa) in the source and analyser zone of the mass spectrometry instruments, scattering of ions occurs during their acceleration. This scattering process causes the energy spread of the ion beam and it is recognized as a peak tail in the mass spectra. In the case of ^90^Sr ion beam detection, undesired interference exists because of the peak tailing of the major abundant ^88^Sr isotope. To depict this interference the abundance sensitivity term is used and defined as:

1 $$Abundance\,sensitivity=\frac{{\rm{Ion}}\,{\rm{intensity}}\,{\rm{at}}\,\mathrm{mass}\,90}{{\rm{Ion}}\,{\rm{intensity}}\,{\rm{at}}\,\mathrm{mass}\,88}$$

For reliable ^90^Sr analysis in environmental samples, abundance sensitivity in the range of 10^−7^ to 10^−11^ is required.

Although, there is a new trend to utilize mass spectrometry instruments for ^90^Sr analysis, those methods are neither well established nor routinely used. Accreditation to an internal standard applying mass spectrometry method for ^90^Sr determination has not been well documented. The main reason for the less development is due to interdisciplinary challenges of this method. Traditionally, the determination of ^90^Sr is carried out by radio-chemists with a strong chemistry and nuclear measurement background while the application of mass spectrometry instrument is the domain of mass spectrometry experts with strong physics and/or engineering background.

Considering the need of rapid and precise ^90^Sr determination after the Fukushima nuclear accident, a ^90^Sr analysis method was developed using the Phoenix X62 TIMS in the laboratory of the National Institutes for Quantum and Radiological Science and Technology (QST), Japan. During the validation process of this new method, detection limit, abundance sensitivity (^90^Sr/^88^Sr), accuracy and precision were determined with reference materials e.g. IRMM-426 (wild berry) and NIST-4354 (freshwater lake sediment) assay proving that the method is suitable for environmental level ^90^Sr analysis^6^.

To establish a higher level of quality assurance, it is essential to participate in a proficiency test controlled by an internationally recognised reference laboratory. To fulfil this standard, our laboratory participated in the IAEA-TEL-2017 world-wide open proficiency test organized by the International Atomic Energy Agency. For the first time, an inorganic mass spectrometry method was employed in a proficiency test for ^90^Sr analysis, which is the exclusive territory of radiometric methods.

## Results

In 2017, the IAEA-TEL-2017 World-Wide Open Proficiency Test was organized by the Reference Materials Group of the IAEA Terrestrial Environment Laboratory, Seibersdorf, Austria, in order to monitor the analytical capabilities and reveal gaps in analytical methodologies of different radioanalytical laboratories^13^. For this purpose, five different samples spiked with known amounts of target radionuclides were distributed. Three tap water samples (Sample 1, 2 and 3) each around 500 g, one milk powder (Sample 4) around 180 g and one Ca-carbonate from spring water (Sample 5) around 40 g were allocated. The samples were prepared with various alpha-, beta- and gamma-emitter natural (radionuclides of ^238^U and ^232^Th decay series) and anthropogenic (isotopes of Cs, Ba, Zr, Tc, Mo, Ru, H, I, Ce, La, Nd, Nb, Np, Sr) radionuclides but our laboratory has interest only in the ^90^Sr determination.

For ^90^Sr determination, Sample 1, 3 and 4 were spiked with ^90^Sr. Sample 3 was a quality control sample with described radionuclide content. The details of the samples analysed are summarised in the Table 1.

**Table 1:** Features of the proficiency test samples.

Sample	Stable Sr^a^ ng·g^−1^	^90^Sr ref. value Bq kg^−1^ (fg g^−1^)^d^	^90^Sr/^88^Sr ratio	Stable Zr^a^ pg·g^−1^	Zr DF
Sample-1 Tap water	39.4 ± 0.9	11.2 ± 0.3 (2.2 ± 0.1)	6.47 × 10^−8^	40 ± 4	1.6 × 10^5^
Sample-3 Tap water QC^b^	39.4 ± 0.9	23.7 ± 0.3 (4.6 ± 0.1)	1.37 × 10^−7^	40 ± 4	8 × 10^4^
Sample-4 Milk powder	2.5 ± 0.1^c^	99.9 ± 5 (19.5 ± 1.1)	9.04 × 10^−9^	<10	<5 × 10^3^

^a^triplicate measurement, ^b^quality control, ^c^(µg·g^−1^), ^d^k = 1.

As discussed in the introduction, Sr and Zr concentration can affect the reliability of ^90^Sr analysis using mass spectrometry instruments. Naturally, the water contains less amounts of trace elements compared to any kind of mammal milk. Therefore, the water sample contains significantly lower Sr concentration (39.4 ng g^−1^) than the concentrated milk powder (2.5 µg g^−1^). In the water samples, the ^90^Sr concentrations were in the same range, 23.7 Bq kg^−1^ (4.6 fg g^−1^) and 11.2 Bq kg^−1^ (2.2 fg g^−1^) respectively. In the milk powder it was higher, 99.9 Bq kg^−1^ (19.5 fg g^−1^), although it corresponds to 13 Bq kg^−1^ (2.5 fg g^−1^) in the original milk sample since the average dry matter content of a raw cow milk is around 13%^14^. Thus, stable Sr and ^90^Sr concentrations were different in samples, ^90^Sr/^88^Sr isotope ratio also differed and varied in the range from 1.37 × 10^−7^ to 9.04 × 10^−9^. The measured ^90^Sr/^88^Sr isotope ratios and the calculated ^90^Sr concentrations along with the detection limits are shown in Tables 2–4.

**Table 2:** Results of TIMS measurements of the quality control tap water.

Sample	^90^Sr/^88^Sr isotope ratio	^90^Sr activity concentration Bq·kg^−1^	DC^b 90^Sr activity concentration Bq·kg^−1^	Detection Limit Bq·kg^−1^
Water-QC-1	1.38 ± 0.03 × 10^−7^	23.5 ± 0.4^a^	23.8 ± 0.4^a^	0.04
Water-QC-2	1.37 ± 0.02 × 10^−7^	23.4 ± 0.3	23.7 ± 0.3	0.04
Water-QC-3	1.39 ± 0.02 × 10^−7^	23.6 ± 0.3	23.9 ± 0.3	0.04
Water-QC-4	1.37 ± 0.02 × 10^−7^	23.3 ± 0.3	23.6 ± 0.3	0.04
**Mean DC value: 23.8** ± **0.3**				
**Reference value: 23.7** ± **0.3**^**a**^				

^a^k = 1. ^b^Decay corrected.

**Table 3:** Results of TIMS measurements of proficiency test tap water.

Sample	^90^Sr/^88^Sr isotope ratio	^90^Sr activity concentration Bq·kg^−1^	DC^b 90^Sr activity concentration Bq·kg^−1^	Detection Limit Bq·kg^−1^
Water-1	6.78 ± 0.06 × 10^−8^	11.6 ± 0.4^a^	11.8 ± 0.4^a^	0.04
Water-2	6.71 ± 0.11 × 10^−8^	11.5 ± 0.4	11.6 ± 0.4	0.04
Water-3	6.76 ± 0.11 × 10^−8^	11.5 ± 0.4	11.7 ± 0.4	0.04
Water-4	6.73 ± 0.10 × 10^−8^	11.5 ± 0.3	11.7 ± 0.3	0.04
Water-5	6.71 ± 0.10 × 10^−8^	11.5 ± 0.4	11.6 ± 0.4	0.04
**Mean DC value: 11.7** ± **0.4**				
**Reference value: 11.2** ± **0.3**^**a**^				

^a^k = 1. ^b^Decay corrected.

**Table 4:** Results of TIMS measurements of proficiency test milk powder.

Sample	^90^Sr/^88^Sr isotope ratio	^90^Sr activity concentration Bq·kg^−1^	DC^b 90^Sr activity concentration Bq·kg^−1^	Detection Limit Bq·kg^−1^
Milk powder-1	8.77 ± 0.51 × 10^−9^	95.3 ± 6.6^a^	97.0 ± 6.7	2.3
Milk powder -2	8.82 ± 0.43 × 10^−9^	95.8 ± 5.6	97.5 ± 5.7	2.3
Milk powder -3	8.88 ± 0.48 × 10^−9^	96.5 ± 5.5	98.2 ± 5.6	2.3
Milk powder -4	8.74 ± 0.49 × 10^−9^	95.0 ± 4.1	96.6 ± 4.2	2.3
Milk powder -5	9.04 ± 0.50 × 10^−9^	98.2 ± 4.1	99.9 ± 4.4	2.3
**Mean DC value: 97.9** ± **5.5**				
**Reference value: 99.9** ± **5**^**a**^				

^a^k = 1. ^b^Decay corrected.

The quality control tap water samples were measured four times while the test samples five times. In the report submitted to IAEA, the mean decay corrected results were reported, 11.7 ± 0.4 Bq kg^−1^ for the tap water samples and 97.9 ± 5.5 Bq kg^−1^ for the milk powder (k = 1). The range of TIMS measurement results were very narrow, varied in the range of 11.6–11.8 Bq kg^−1^ for tap water samples and 96.6–99.9 Bq kg^−1^ for milk powder samples. Each measurement produced results with excellent accuracy and precision compared to the reference values of 11.2 ± 0.3 (2.2 ± 0.1 fg g^−1^) and 99.9 ± 5 Bq kg^−1^ (19.5 ± 1.0 fg g^−1^), respectively. These results demonstrated the robust analytical capacity of TIMS for ^90^Sr analysis.

### Statistical analysis

#### Water

**Figure 1:**
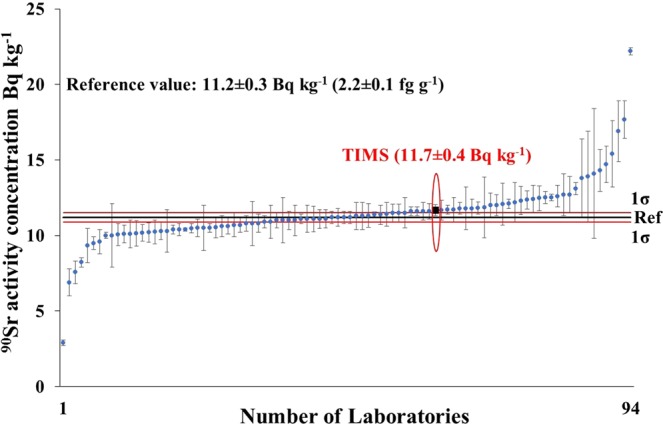
Reported results and evaluation parameters of the ^90^Sr in proficiency test tap water sample.

The reported results of the proficiency test were published in the evaluation report^13^. The results of tap water sample (Sample 1) are shown in Fig. 1. Totally 94 laboratories were involved and 75 (80%) laboratory performances were ‘accepted’ while 6 (6%) were warned (not acceptable precision) and 13 (14%) were ‘not accepted’. The ranges of the results were wide, from 2.9 to 22.2 Bq kg^−1^. The TIMS result (marked with black quadrat) proved that the new TIMS method is competitive with radiometric methods for the measurement of ^90^Sr in water samples.

**Figure 2:**
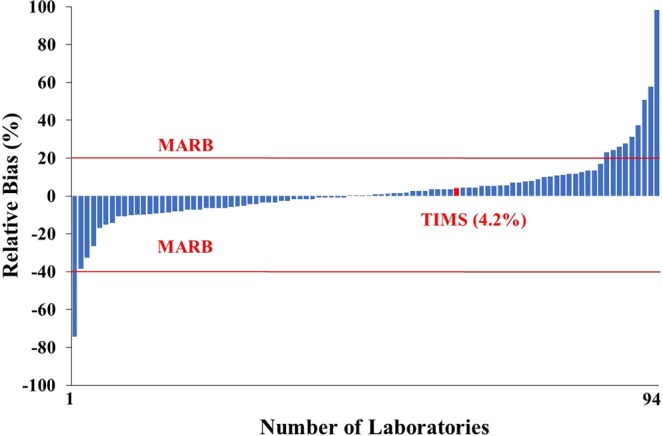
Relative bias (accuracy) of ^90^Sr results in the proficiency test tap water sample.

TIMS result (11.7 Bq kg^−1^) was slightly higher than the reference value (11.2 Bq kg^−1^) thus the relative bias (RB) statistical parameter, calculated by Eq. 3, was quite good, 4.2% as illustrated in Fig. 2, where the range of the RB was quite wide and varied from −74.2 to 98.2%. The relative bias value of TIMS measurement was significantly lower than the maximum acceptable relative bias (MARB) for tap water samples (20%) thus the accuracy of TIMS measurement were ‘accepted’ (acceptance criteria of the accuracy: RB ≤MARB).

**Figure 3:**
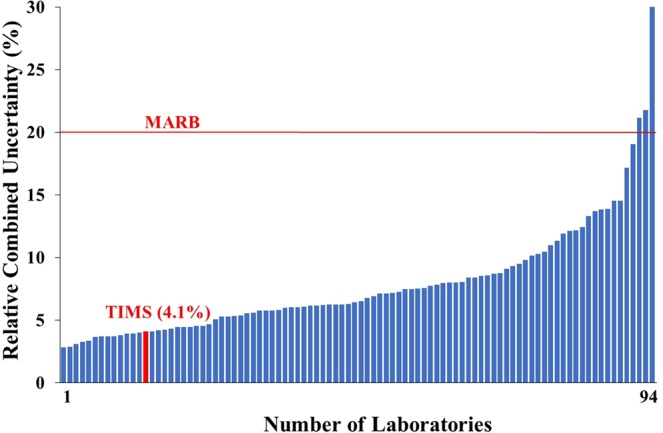
Relative combined uncertainty (precision-P value) of ^90^Sr results in the proficiency test tap water sample.

To satisfy the performance criteria of the precision of an applied measurement method, the reported results must overlap with the reference value within their uncertainties. Additionally, the precision (P) must be below (or equal to) the MARB and over (or equal to) the relative bias (k = 2.58) as interpreted in Eq. 4^15^. In this study, the uncertainty of TIMS measurements was 3.1%, the calculated P value 4.1% thus the precision attained was satisfactory and certified as ‘accepted’. The precisions of the tap water measurements are shown in Fig. 3. The precision of the measurements fluctuated between 2.8 and 30.6% and only in three cases was too high to be accepted. In the Fig. 3 only the MARB can be seen as performance criteria since the RB varies individually thus not presentable. In three more cases, the precision below the MARB level, was not accepted because of high RB (RB ˃2.58 × P) of the measurement results (which is not shown in Fig. 3). The major reason of failure in the proficiency test is the low accuracy (high RB) of the values reported by the participant laboratories as can be seen from the results of Figs 2 and 3.

**Figure 4:**
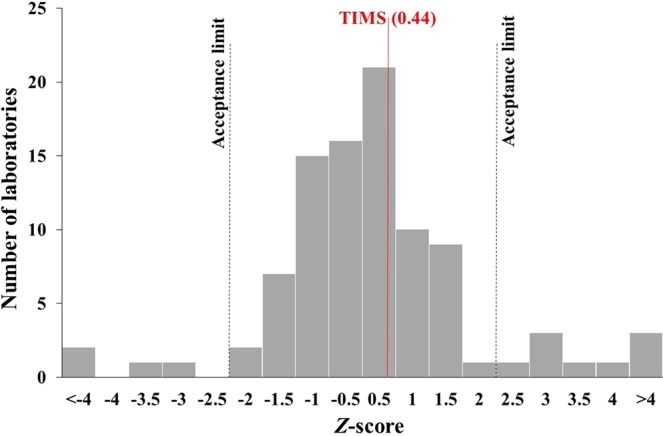
Z-score histogram of ^90^Sr measurement of the proficiency test tap water samples.

The *z*-score statistics has been used to evaluate the performances of the participant laboratories and are shown in Fig. 4. The *z*-score calculation was carried out using the robust standard deviation described in Eqs 5 and 6. As it can be seen from the calculation method, the z-score value does not give exact information about the precision only the accuracy. Moreover, the calculated *z*-score is a relative parameter, as robust standard deviation originates from the reported results and is affected by the performance of the participant laboratories. The reported results with a higher *z*-score of 2 not acceptable. The range of *z*-scores was from −7.7 to 10.3. Four laboratories reported exactly the same value as the reference one (*z*-score = 0) and seven more laboratories presented values with almost the same *z*-score as in the range from −0.01 to 0.01. In our case, the *z*-score of 0.44 was in the range from −0.5 to 0.5 which is quite impressive since only 37 laboratories of the 94 were able to accomplish this level of accuracy.

### Statistical analysis

#### Milk powder

**Figure 5:**
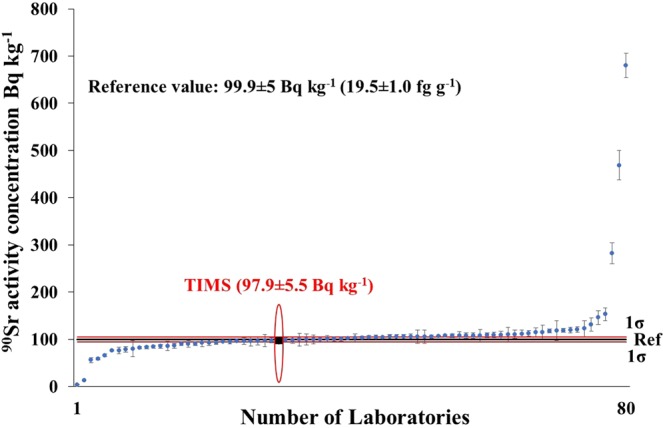
Reported results and evaluation parameters of ^90^Sr in proficiency test milk powder sample.

The results of the milk powder sample (Sample 4) are shown in Fig. 5. Totally 80 laboratories were involved, 54 (67.5%) laboratories gained the “accepted” status while 26 (32.5%) laboratories were not able to fulfil the performance criteria of proficiency test (MARB = 15%). The ranges of the reported results were also very wide, from 3.9 to 680 Bq kg^−1^. Compared to tap water results, reliable ^90^Sr analysis in milk powder seems a more challenging task.

**Figure 6:**
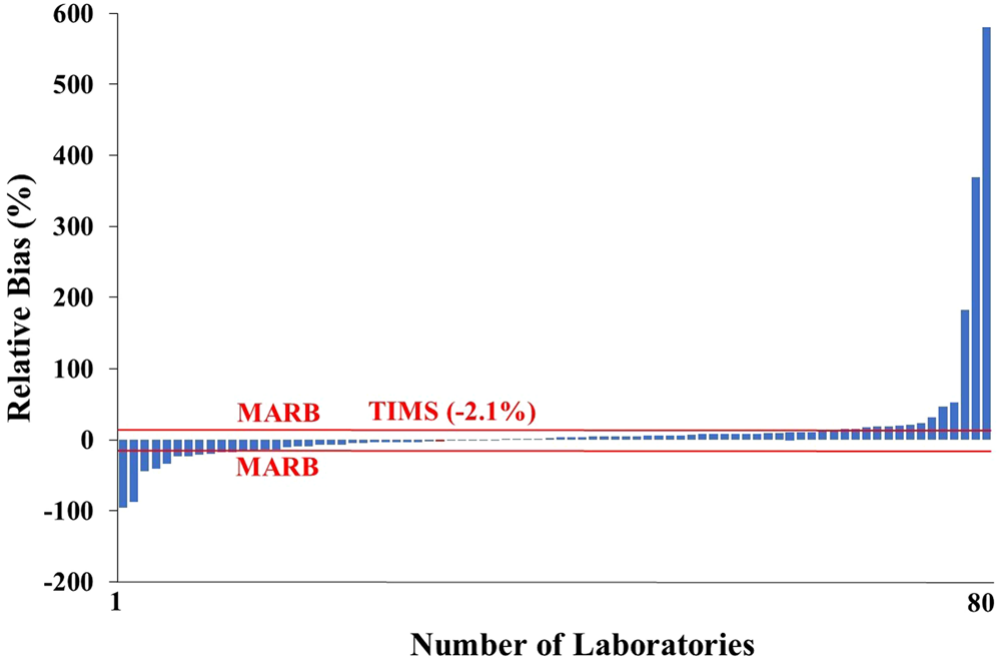
Relative bias (accuracy) of ^90^Sr results in the proficiency test milk powder sample.

The TIMS result is marked here with a black quadrat and confirmed that this novel TIMS method is competitive with radiometric methods to determine ^90^Sr in milk powder samples. In this case, TIMS result (97.9 Bq kg^−1^) was slightly lower than the reference value (99.9 Bq kg^−1^) thus the relative bias was also very good, −2.5% as presented in Fig. 6 where the range of the RB was very wide, from −96.1 to 580.7%. Since the relative bias of the TIMS result was significantly lower than the MARB value (15%), the “accepted” certificate was obtained for the accuracy.

**Figure 7:**
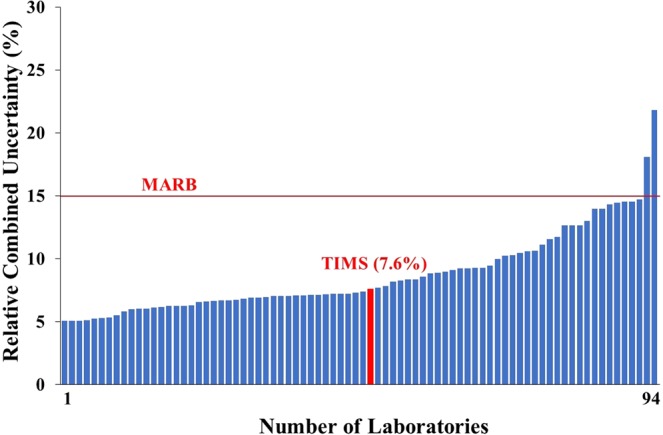
Relative combined uncertainty (precision-P value) of ^90^Sr results in the proficiency test milk powder sample.

The uncertainty of this TIMS measurement was 5.7% and the calculated P value was 7.6% therefore the precision of the TIMS method complied with the standards assigned by the IAEA proficiency test. The precision of the milk powder measurement is given in Fig. 7 and it was found to range between 5.1 and 21.8%. There were only two measurements reported where the precisions were over the 15% MARB as can be seen in Fig. 7 and 19 more measurements (not presented in Fig. 7) where the RB was too high (low accuracy) to be covered by the expanded relative combined uncertainty (2.58 × P).

**Figure 8:**
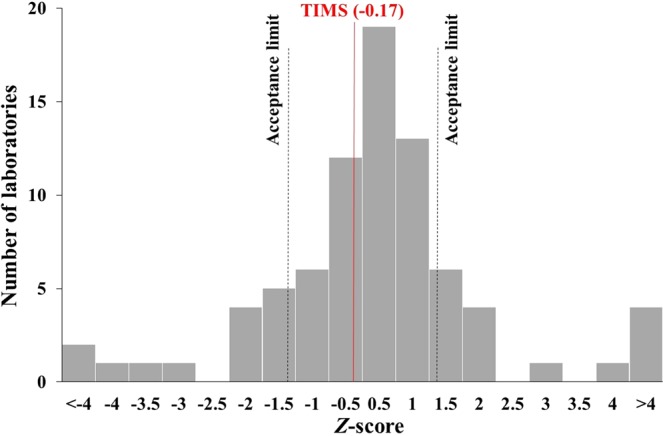
Z-scores histogram of ^90^Sr measurement results of the proficiency test milk powder sample.

The calculated *z*-scores of the milk powder measurements are presented in the Fig. 8. Here the *z*-score acceptance limit (1.1) was much lower than in case of the water sample (2) because the MARB level for milk powder (15%) was lower, than for tap water (20%). In this case, the range of *z*-scores was quite wide, from −7.7 to 46.7. However, the *z*-score values of 31out of 80 laboratories reported were in the range from −0.5 to 0.5 as well as our laboratory with the excellent result of −0.17.

## Discussion

### Precision of the TIMS method

TIMS is a highly precise instrument to measure accurately stable isotope ratios. For instance, stable Sr isotope ratios can be determined with a relative standard deviation of around 0.003%^16,17^. This precision performance is not possible for ^90^Sr analysis because the sample amount of ^90^Sr loaded onto a single degassed zone refined Re filament is very low, only a few fg^6^. Therefore, the generated ^90^Sr ion beam is quite weak and only few counts are detectable with a relatively high measurement error. In case of tap water sample (Sample 1), around 400 ng stable strontium was loaded along with 20 fg ^90^Sr, while for milk powder samples (Sample 4) around 1000 ng stable strontium was loaded along with 8 fg ^90^Sr. Consequently, the ^90^Sr beam intensity was higher for tap water samples, resulting in better relative standard deviation of ^90^Sr/^88^Sr isotope ratio (~2%) than for the milk powder (~5%) as can be seen in Tables 3 and 4. The total number of cycles measurement were 50 for both samples, consequently the final result was derived from the average of 50 measurement cycles.

The detection capacity limit of the Faraday cups during TIMS measurement for abundant ^88^Sr or ^86^Sr peaks play a significant role that needs special attention. At the beginning, a direct measurement of the ^88^Sr peak on a Faraday cup and simultaneously ^90^Sr on a Daly ion-counter was tested. ^88^Sr ion beam detection was not possible over 10 V using Faraday cup with resistor of 10^11^ Ω and the ^90^Sr ion beam remained undetectable on the Daly ion-counter if the ^90^Sr/^88^Sr isotope ratio was lower than 10^−8^. Since there was no way to expand the detection range of the Faraday cups, a less abundant Sr isotope, ^86^Sr was used to detect the reference ion beam instead of ^88^Sr. In Sr element the abundance of ^88^Sr is 82.58%, whereas the abundance of ^86^Sr is 9.86%. Moreover, the ^86^Sr/^88^Sr isotope ratio (0.1194) is a naturally stable ratio with fluctuation less than 0.1%^18^. Considering that the uncertainty of the ^90^Sr determination is more than 1% for the TIMS method, the uncertainty of ^86^Sr/^88^Sr isotope ratio has to be compared always with the overall measurement uncertainty for all kinds of environmental samples. The ^87^Sr isotope or ^87^Sr/^88^Sr isotope ratio cannot be utilized due to the radiogenic origin of the ^87^Sr (decay product of the ^87^Rb).

### Detection limit of the TIMS method

The detection limit (DL) of ^90^Sr analysis for radiometric methods is affected by two major parameters. One is the background count rate and second the sample amount. Basically, the background count rate is a constant, caused by background radiation derived from ^40^K, U- and Th-decay series (these natural radionuclides occurs in building materials, metal parts of the instrument, e.g. lead shield, etc.) and electric noise of the measurement system^19^. With increments of the sample intake, ^90^Sr concentration is increased in the analyte and the DL can be improved. However, the limitation lies in expenditure (necessity of more chemicals, resins, etc.) and increase in preparation time along with the sample amount.

There are two affecting parameters also for the mass spectrometry method. One is the ^90^Sr/^88^Sr abundance sensitivity, corresponding to a background effect of the peak tail of ^88^Sr and electric noise of the instrument. The abundance sensitivity is a kind of quality of a given mass spectrometry instrument which may decline with time as the pressure degrades mainly in the analyser house. However, the abundance sensitivity must be considered as a constant and needs to be checked regularly as a protocol of quality control.

The other parameter is not the sample intake itself but the stable Sr concentration of sample. For example, if one litre water and milk have the same level of ^90^Sr contamination, the ^90^Sr/^88^Sr isotope ratio will be higher in the water sample than in the milk sample because water has a significantly lower stable Sr concentration than the milk. Therefore, if ^90^Sr/^88^Sr abundance sensitivity is a constant, the detection limit will be lower for water than milk samples. In this proficiency test, as it can be observed in Table 2, tap water (39.4 ± 0.9 ng g^−1^) had two magnitudes lower stable Sr concentration than milk powder (2.5 ± 0.1 µg g^−1^). This explains the detection limit data in Tables 3 and 4, where the detection limit is two magnitudes lower for the tested water (0.04 Bq kg^−1^) than for milk powder sample (2.3 Bq kg^−1^). Consequently, mass spectrometry is competitive with radiometric method if the stable Sr concentration is in the range of ng g^−1^ in environmental samples, such as in fresh water samples.

### Sample intake

The stable Sr concentration also influences the sample size that is necessary for ^90^Sr analysis with TIMS. Typically, 400 ng of stable Sr loaded on the filament is adequate for reliable analysis but larger sample amounts result in a more controllable beam and more measurement cycles. Obviously, to get the required amount of stable Sr, more water sample was used than milk powder for ^90^Sr analysis. Counting with some strontium loss during the sample preparation and separation, the sample intake was calculated with 2000 ng stable strontium. This stable strontium amount corresponded to 50 mL water sample and 1 g milk powder. The homogenous distribution of the added ^90^Sr in water sample is not an issue but in case of the milk powder non-homogeneity may occurs. To decrease the uncertainties derived from non-homogeneity, 50 g milk powder sample was recommended by the organizers of the proficiency test as a minimum sample intake. Since the sample preparation and strontium separation procedures of the TIMS method is optimized for 2000–4000 ng stable strontium, the risk was taken and only 1 g milk powder sample was digested to analyze ^90^Sr. As our measurement results demonstrated, the homogeneity of ^90^Sr in the milk powder sample was excellent and 1 g sample represented ^90^Sr distribution perfectly.

The sample homogeneity and stability are fundamental requirements for reference materials and proficiency test materials. In case of homogeneity test, the advantage of the mass spectrometry method is to be recognized. For example, to test the homogeneity of ^90^Sr in soil matrix, 100 mg or less sample amount is sufficient for reliable ^90^Sr analysis using the TIMS instrument, however the detection limit must be considered.

### Limitations of the proficiency test and quality control

**Figure 9:**
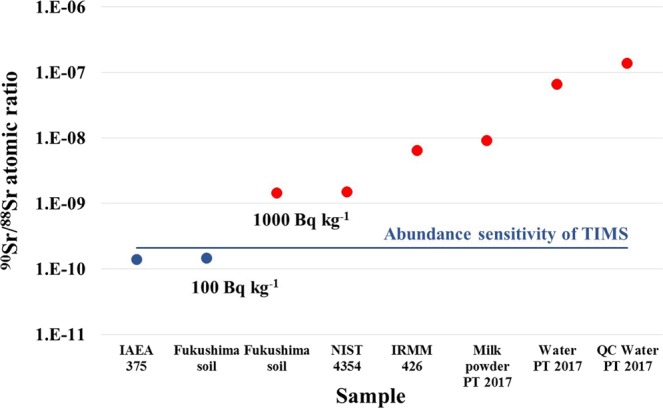
The ^90^Sr/^88^Sr isotope ratio in different reference materials and samples of the IAEA proficiency test (2017) (decay correction date is November 15, 2017).

The TIMS method demonstrated a very precise and accurate ^90^Sr measurement capacity but the limitations of the proficiency test must be considered. In case of the mass spectrometry methods, both the ^90^Sr activity concentration and the ^90^Sr/^88^Sr isotope ratios are important. As it can be observed in Fig. 9, ^90^Sr/^88^Sr isotope ratio of the water and milk powder samples distributed for the IAEA proficiency test are relatively high, varies between 1.4 × 10^−7^ and 9.0 × 10^−9^ therefore ^90^Sr detection was rendered possible using TIMS. Moreover, the Zr interference was not a real challenge since Zr concentrations in the samples were quite low (pg g^−1^ range). As Fig. 9 presents, soil sample analysis is more demanding due to higher concentrations of stable Sr (µg g^−1^ range) and Zr (µg g^−1^ range) concentration in soil samples. In case of Fukushima soil samples wherein stable Sr concentration is over 100 µg g^−1^, ^90^Sr is not detectable around 100 Bq kg^−1^ but could be confidently measured at around 1000 Bq kg^−1^. Consequently, TIMS ^90^Sr measurement capacity is out of range to use the most preferred and frequently used IAEA-375 reference material (Chernobyl affected soil sample) for internal quality control.

**Figure 10:**
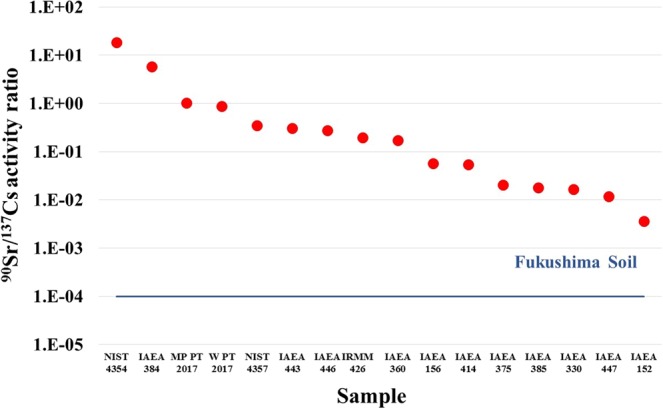
The ^90^Sr/^137^Cs activity ratio in different reference materials and samples of the IAEA proficiency test (2017).

As Fig. 9 demonstrates there are adequate reference materials containing ^90^Sr for mass spectrometry analysis (wide range of ^90^Sr/^88^Sr isotope ratios and Zr concentration) but interestingly for radiometric analysis it is lacking, considering the characteristics of the Fukushima accident. Fukushima accident affected environmental samples are mainly contaminated with volatile beta emitter fission products such as, I, Te and Cs isotopes. The contamination of non-volatile (Sr, Ru isotopes etc.) and refractory elements (Pu, U isotopes etc.) are very low in comparison because the primary concrete containment system mounted at the Fukushima Daichi reactors and the reactor pressure vessels were not damaged significantly by hydrogen explosions^20–22^. The ^90^Sr (and ^89^Sr) is a pure beta emitter radionuclide and beta particles have continuous spectrum unlike gamma rays therefore efficient separation of interfering beta emitter radionuclides is crucial before ^90^Sr measurement with radiometric instruments (every fission product is a beta emitter as well as gamma emitter except ^90^Sr and ^89^Sr). In Fukushima environmental samples the ^137^Cs (*T*_*1/2*_ = 30.2 y) has the highest significance for long-term radioactive contamination^23^. The ^90^Sr/^137^Cs activity ratio in Fukushima contaminated samples is around 1 × 10^−4^ which corresponds to a Cs decontamination factor of 10^5 ^^24^. As can be seen in Fig. 10, reference materials with this ^90^Sr/^137^Cs activity ratio are not available, wherein presence of ^137^Cs significantly dominates ^90^Sr. The ^90^Sr/^137^Cs activity ratio in certified reference materials varies between 3.6 × 10^−3^ and 1.8 × 10^1^ (only the IAEA-152 milk sample was in the range of 10^−3^ but acquisition of this sample is not possible as it is out of stock). Consequently, applicable internal quality control is not possible and valid proficiency tests are not available for laboratories where ^90^Sr determination is carried out in Fukushima samples using radiometric methods. One possible way to solve this problem, until the production of proper reference materials, is the cooperation of laboratories where mass spectrometry methods are applied for ^90^Sr analysis and selected Fukushima samples could be determined in both laboratories for the performance estimation (inter-laboratory comparison).

## Materials and Methods

### Sample preparation

50 mL water sample was evaporated and dissolved with 5 mL 8 M HNO_3_. 1 g milk powder was ashed at 600 °C and dissolved with 5 mL 8 M HNO_3_.

For HNO_3_ solution preparation, analytical grade Tamapure AA-100 reagents (68% w/w) and Milli-Q2 purified water (>*18* MΩ cm at 25 °C) were used.

### Strontium separation

For strontium separation 0.5 mL DGA and Sr extraction chromatography resin of 100–150 μm particle size (Eichrom Technologies Inc, USA) packed into polypropylene gravity columns (Muromac, Japan; size, 42 mm in length and 5 mm in diameter) and washed with 10 mL H_2_O and preconditioned with 5 mL 8 M HNO_3_ with a flow rate of ~0.2 mL min^−1^. The samples dissolved in 5 mL 8 M HNO_3_ were loaded into the prepared columns and rinsed with 3 mL 8 M HNO_3_ and 3 mL 3 M HNO_3_. Finally, the Sr was stripped from Sr resin with 3 mL 0.05 M HNO_3_.

### Sample loading

The 0.05 M HNO_3_ solution was evaporated to about 0.1 mL and mixed with 0.5 ml HNO_3_ and 0.5 mL H_2_O_2_. This solution was boiled for 10 minutes and evaporated to dryness. Thereafter, the residue containing Sr was dissolved in 0.25 ml 1 M HNO_3_ and evaporated to a tiny drop. The Sr sample was loaded onto a degassed single rhenium filament (99.999%) along with one microliter TaF_5_ activator.

The detailed information of reagents and materials, sample preparation, strontium separation and ^90^Sr analysis using TIMS are given elsewhere^6,12^.

### Instruments

**Figure 11:**
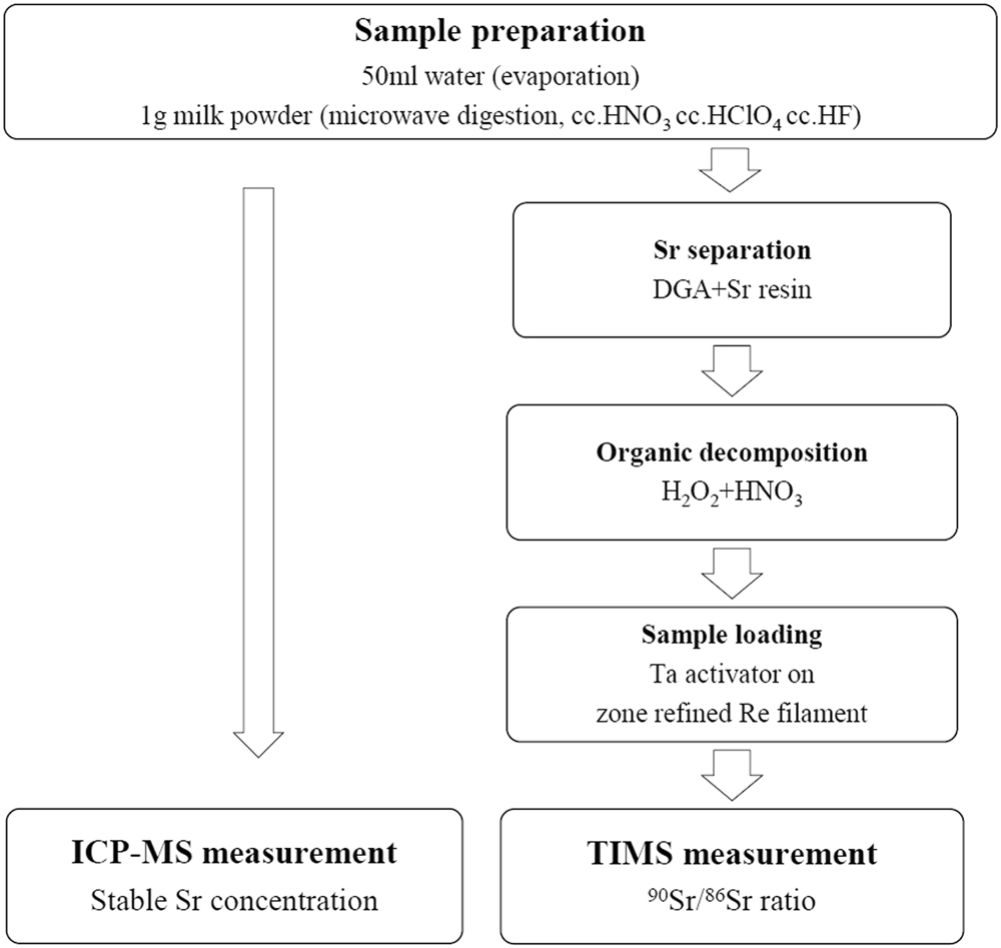
The schematic diagram of ^90^Sr analysis method using mass spectrometry instruments.

**Figure 12:**
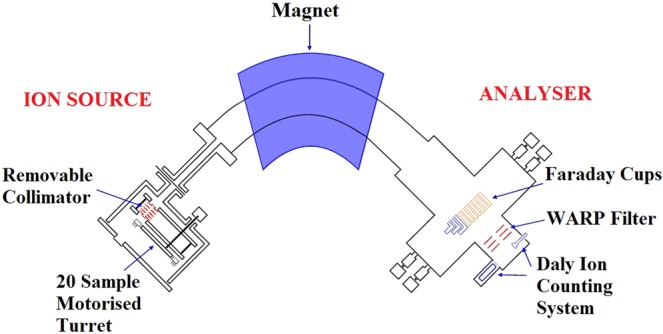
The layout of the Phoenix X62 TIMS.

**Figure 13:**
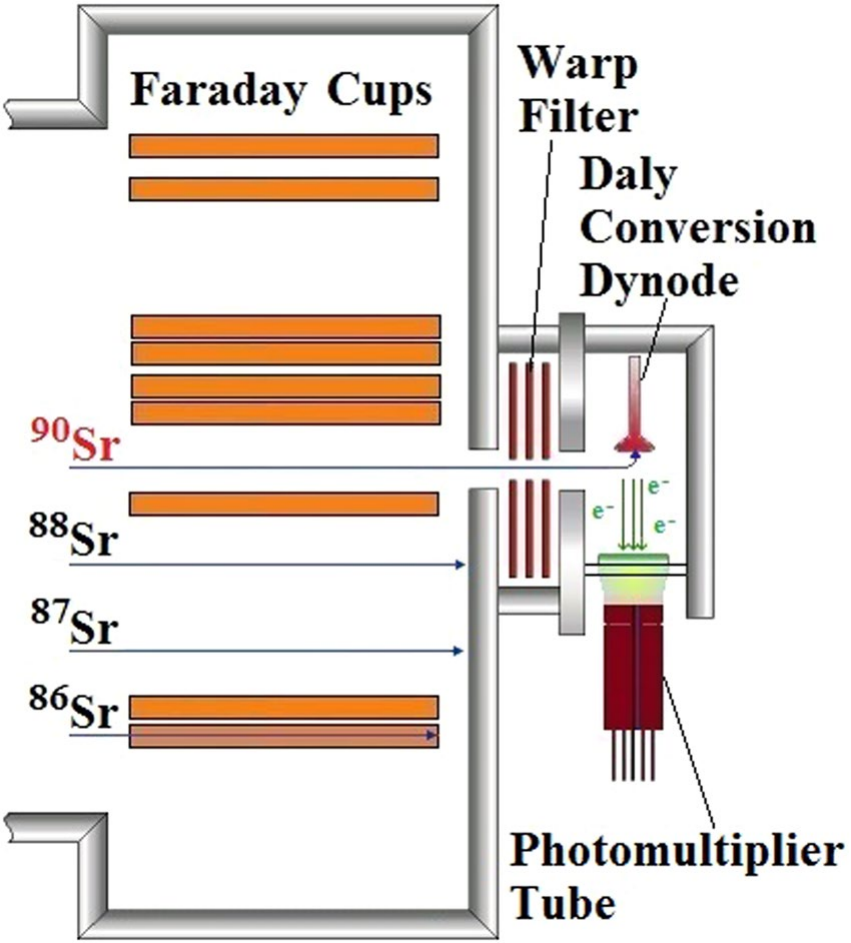
Multi-collector assembly and detector set up of the Phoenix62 TIMS for ^90^Sr analysis.

The TIMS instrument used are described elsewhere^6^ but schematic diagram of the ^90^Sr measurement method (Fig. 11), layout of the Phoenix X62 TIMS (Fig. 12) and ^90^Sr detection in the TIMS analyser section (Fig. 13) are presented here:

During the measurement, ^86^Sr ion beam is detected, while ^88^Sr ion beam is not collected by any Faraday cups and directed into the analyser wall. The ^90^Sr/^88^Sr isotope ratio is calculated from the measured ^90^Sr/^86^Sr ratio^6^. With this method, ^90^Sr ion beam is detectable on the Daly ion-counting detector in samples where ^90^Sr/^88^Sr isotope ratio is in the range of 10^−9^ and 10^−10^ as it occurs in many environmental samples.

For stable strontium concentration determination, Agilent-8800 inductively coupled plasma mass spectrometer (Agilent Technologies, USA) was used. The detailed strontium analysis method is described elsewhere^25^.

### Interference removal of ^90^Zr

Owing to the evaporation temperature and ionization potential difference between Sr (~1380 °C and 5.7 eV) and Zr (~1900 °C and 6.6 eV), Zr separation can be accomplished during the thermal ionization process, however to reach higher decontamination level (10^8^ to 10^11^), additional chemical separation of Zr is necessary. Applying two consecutive DGA and Sr resin extraction chromatography separations on environmental samples prior to the TIMS measurement, Zr decontamination factor of 10^11^ can be accomplished^6,12^.

### Abundance sensitivity

The abundance sensitivity of the Phoenix X62 TIMS is 2.1 × 10^−10^, which is excellent due to very stable ion-beam generation via thermal ionization and ultra-high vacuum condition in the source (10^−8^ Pa or lower) and the analyzer (10^−9^ Pa or lower). The abundance sensitivity of the TIMS has been regularly checked and determined measuring NIST-987 standard reference material without any traces of ^90^Sr^6^. The NIST-987 has been purified by extraction chromatography to avoid ^90^Zr interference.

### ^90^Sr activity concentration calculation

The ^90^Sr activity concentration was calculated as the follows:

2 $${A}_{90}={R}_{m}\times {C}_{Sr}\times {N}_{A}\times \frac{{{\rm{NA}}}_{88}}{{{\rm{M}}}_{Sr}}\times \frac{ln2}{{T}_{1/2}}$$

where *A*_90_ is the ^90^Sr activity concentration (Bq·kg^−1^), *R*_*m*_ is the measured ^90^Sr/^88^Sr isotope ratio, *C*_*Sr*_ is the stable Sr concentration of the sample (mg·kg^−1^), *N*_*A*_ is the Avogadro constant, *NA*_88_ is the natural abundance of ^88^Sr (0.8258), *M*_*Sr*_ is the atomic mass of the Sr element (87.62 amu), *T*_*1/2*_ is the half-life of the ^90^Sr (9.1 × 10^8^ s).

### Statistical calculation

The statistical calculations discussed are presented elsewhere^15,26^ but some important calculations are given here:

3 $$RB=\frac{{A}_{m}-{A}_{ref}}{{A}_{ref}}\times 100 \% $$

Acceptance criteria of accuracy

$$RB\le {\rm{MARB}}$$

where, *RB* is the relative bias, *A*_*m*_ is the reported ^90^Sr activity concentration (Bq·kg^−1^) and *A*_*ref*_ is the reference ^90^Sr activity concentration (Bq·kg^−1^), MARB is the maximum acceptable relative bias.

4 $$P=\sqrt{{(\frac{{u}_{m}}{{A}_{m}})}^{2}+{(\frac{{u}_{ref}}{{A}_{ref}})}^{2}}\times 100 \% $$

Acceptance criteria of precision

$$P\le {\rm{MARB}}\,{\rm{and}}\,RB\le 2.58\times {\rm{P}}$$

where, *P* is the precision of the measurement, *u*_*m*_ is the combined uncertainty (k = 1) of the reported ^90^Sr activity concentration (Bq·kg^−1^) and *u*_*ref*_ is the combined uncertainty (k = 1) of the reference ^90^Sr activity concentration (Bq·kg^−1^).

5 $$z=|\frac{{A}_{m}-{A}_{ref}}{Robust\,sd}|$$

where, *z* is the *z*-score value, *A*_*m*_ is the reported ^90^Sr activity concentration (Bq·kg^−1^) and *A*_*ref*_ is the reference ^90^Sr activity concentration (Bq·kg^−1^), *Robust sd* is the robust standard deviation (Bq·kg^−1^).

The robust standard deviation was calculated as follows:

6 $$Robust\,sd=1.483\times {\rm{median}}\,{\rm{of}}\,|{A}_{m}-{A}_{ref}|$$

## Conclusion

The results of this work demonstrate that our scientific efforts, to push the boundaries of inorganic mass spectrometry into a higher level, were successful. This is the first time that an inorganic mass spectrometry method fulfilled the requirements of a proficiency test in environmental level ^90^Sr determination. Considering the accuracy, precision and detection limit, the new mass spectrometry method is very competitive with the traditional radiometric methods and ready for accreditation. The outstanding sensitivity of Daly ion-counting detector and improved abundance sensitivity of the thermal ionization mass spectrometry widen the application not only stable isotope ratio but also radioactive isotope ratio analysis of radionuclides with a half-life less than 100 years. In case of a nuclear accident when the rapid analysis of ^90^Sr is critical, the usage of this new method is recommended.

The new method needs further improvement to decrease the strontium amount necessary for analysis. This step can significantly reduce the sample intake along with the corrosive acid usage. Furthermore, the separation time can be also shortened promising the most rapid ^90^Sr analysis method in the future. It can also be a useful tool for reference material characterization, where employment of different methods is important for reliable results. Additionally, the low sample intake makes the new method capable to test radionuclide homogeneity in reference material.

Considering the ^90^Sr/^88^Sr isotope ratio, the availability of reference materials is adequate to support the internal quality control process of laboratories where mass spectrometry method used for ^90^Sr analysis. However, from the viewpoint of ^90^Sr/^137^Cs activity ratio, reference material related to the Fukushima accident is not accessible.
